# Supplementary material for: Age at menarche and its association with preschool BMI among girls in Northern Norway
Source: PLoS One. 2025 May 13;20(5):e0322986. doi: 10.1371/journal.pone.0322986 (PMC12074588; doi:10.1371/journal.pone.0322986)
Supplement: S1 File — (DOCX) [file pone.0322986.s001.docx]

# **Supporting information - Declarations**

# **1 Ethics approval and consent to participate**

All participants signed an informed consent form. Those younger than 16 years had to provide additional written consent from a guardian. The study was conducted in accordance with the Declaration of Helsinki. The current study was approved by the regional committee for medical and research ethics at Helse Nord (reference number 213685/REK nord) in January 2021.

## **2 Consent for publication**

Not applicable.

## **3 Availability of data and materials**

The data that support the findings of this study are available from Fit Futures (https://uit.no/research/fitfutures_en#region_843358), but restrictions apply to the availability of these data, which were used under license for the current study and are not publicly available.

## **4 Competing interests**

The authors declare that they have no competing interests.

## **5 Funding**

This study received no funding.

## **6 Authors' contributions**

HLJ and AB share first authorship. HLJ coded all the statistical analyses. HLJ and AB contributed to the conception, analysis and interpretation of the data and drafted the paper, including all the tables and figures. GG and EKE contributed to the data collection, study conception and design, and interpretation and substantially revised the paper. ASF was the principal investigator in Fit Futures, and both ASF and CSN contributed to data collection and study design and substantially revised the paper. All the authors have substantially revised and approved the submitted version.

## **7 Declaration of use of AI tools**

In the final stages of manuscript editing, the Curie software of © 2024 American Journal Experts, LLC, was used to correct spelling mistakes and grammar. The software suggested 22 changes of words (i.e., from “in” to “to”), marked 14 grammatical errors, and suggested 44 changes of punctuation and spacing. Of these suggestions, 18 changes of words were accepted, all grammatical errors were assessed and the sentences changed, and 37 of the punctuation and spacing suggestions were accepted.

## **8 Acknowledgments**

We are greatly thankful to the Fit Futures participants. We are especially thankful for the excellent work provided by the personnel at the Clinical Research Unit of the University Hospital of North Norway and to the study coordinator Annelene Moberg for recruiting the participants.
